# Supplementary material for: Reduction in the Cocoa Spontaneous and Starter Culture Fermentation Time Based on the Antioxidant Profile Characterization
Source: Foods. 2023 Sep 1;12(17):3291. doi: 10.3390/foods12173291 (PMC10487274; doi:10.3390/foods12173291)
Supplement: Supplementary file 1 [file foods-12-03291-s001.zip › foods-2574390-supplementary.pdf]

Table S1: Average values of pH, dissolved oxygen and temperature (°C) in cocoa pulp-bean mass in SF and SC

	Spontaneous Fermentation (SF)			Fermentation with starter culture (SC)		
Sample	pH	OD	Temperature	pH	OD	Temperature
Gua_0h	3.797	5.600	28.47	3.803	0.767	28.63
Gua_24h	3.943	0.833	29.87	3.777	0.400	33.70
Gua_48h	3.877	2.067	32.53	3.847	1.800	34.10
Gua_72h	4.217	2.133	46.27	4.113	2.100	45.80
Gua_96h	4.130	1.500	46.10	3.967	1.733	45.97
Gua_120h	4.260	2.033	42.43	3.887	1.600	44.20
Gua_144h	4.220	0.567	42.33	3.910	1.133	43.90
Gua_168h	4.467	1.367	48.63	4.740	1.700	48.23
Tol_0h	4.020	0.367	27.40	4.050	0.633	27.57
Tol_24h	3.907	0.267	28.27	3.637	1.067	29.33
Tol_48h	3.727	0.367	34.93	3.803	0.300	43.40
Tol_72h	4.250	1.267	44.53	4.127	1.000	43.70
Tol_96h	4.283	1.067	45.43	4.153	0.633	42.17
Tol_120h	4.240	0.267	41.23	4.073	0.200	33.47
Tol_144h	4.260	0.133	38.47	4.027	0.233	32.80
Tol_168h	4.453	0.567	39.23	3.933	0.467	31.70
Cop_0h	4.170	2.733	25.70	4.147	2.933	25.40
Cop_24h	3.643	0.633	28.53	4.043	0.500	28.50
Cop_48h	3.897	1.900	33.30	3.863	1.800	34.13
Cop_72h	4.467	1.767	47.20	4.447	1.867	48.03
Cop_96h	4.570	1.867	45.60	4.310	1.900	45.47
Cop_120h	4.740	1.367	47.57	4.733	1.233	47.97
Cop_144h	4.713	0.833	44.60	4.807	0.767	44.37
Cop_168h	5.067	0.533	47.30	5.010	0.400	47.67

Table S2: Average values of physicochemical parameters of cocoa beans during fermentation (SF and SC)

	Spontaneous Fermentation (SC)					Fermentation with starter culture (SC)				
Sample	pH	Aw	Moisture content	TTA	FI	pH	Aw	Moisture content	TTA	FI
Gua_0h	6.637	0.913	29.473	0.029	0.377	6.353	0.909	33.103	0.020	0.352
Gua_24h	6.577	0.916	38.960	0.026	0.506	6.553	0.906	34.167	0.039	0.857
Gua_48h	6.037	0.927	31.043	0.056	0.720	5.553	0.906	29.570	0.049	0.924
Gua_72h	4.960	0.928	30.140	0.140	1.080	5.117	0.910	35.830	0.078	1.442
Gua_96h	4.613	0.929	32.333	0.196	1.602	4.623	0.911	32.680	0.186	1.873
Gua_120h	4.390	0.931	36.110	0.268	1.708	4.480	0.916	32.797	0.248	1.877
Gua_144h	4.550	0.935	40.257	0.229	1.729	4.527	0.913	39.097	0.258	1.862
Gua_168h	4.483	0.928	36.193	0.255	1.829	4.603	0.915	35.940	0.193	1.903
Tol_0h	6.433	0.963	32.930	0.075	0.361	6.437	0.923	27.913	0.049	0.336
Tol_24h	6.150	0.955	29.847	0.075	0.382	6.187	0.906	30.847	0.052	0.372
Tol_48h	4.950	0.947	29.860	0.160	0.591	5.013	0.895	30.493	0.154	0.581
Tol_72h	4.690	0.933	31.610	0.222	1.108	4.533	0.906	37.483	0.317	1.071
Tol_96h	4.467	0.945	32.317	0.242	1.498	4.333	0.921	36.643	0.376	1.412
Tol_120h	4.500	0.946	23.830	0.278	1.652	4.433	0.931	30.220	0.287	1.486
Tol_144h	4.483	0.954	30.357	0.268	1.698	4.373	0.943	32.067	0.271	1.588
Tol_168h	4.617	0.949	23.390	0.216	1.697	4.340	0.935	31.163	0.297	1.455
Cop_0h	6.780	0.902	29.930	0.026	0.331	6.523	0.909	31.560	0.036	0.301
Cop_24h	6.543	0.899	32.363	0.026	0.340	6.597	0.888	28.060	0.023	0.333
Cop_48h	5.997	0.896	33.977	0.042	0.598	5.167	0.880	28.187	0.121	0.412
Cop_72h	5.753	0.894	33.677	0.052	0.661	4.947	0.884	26.700	0.144	0.780
Cop_96h	4.820	0.904	33.273	0.124	0.966	4.433	0.877	30.713	0.307	0.966
Cop_120h	4.930	0.912	41.057	0.150	1.283	4.767	0.898	33.680	0.183	1.432
Cop_144h	4.787	0.916	37.830	0.167	1.371	4.937	0.902	30.773	0.137	1.510
Cop_168h	4.920	0.904	28.087	0.108	1.359	4.747	0.913	34.183	0.157	1.355

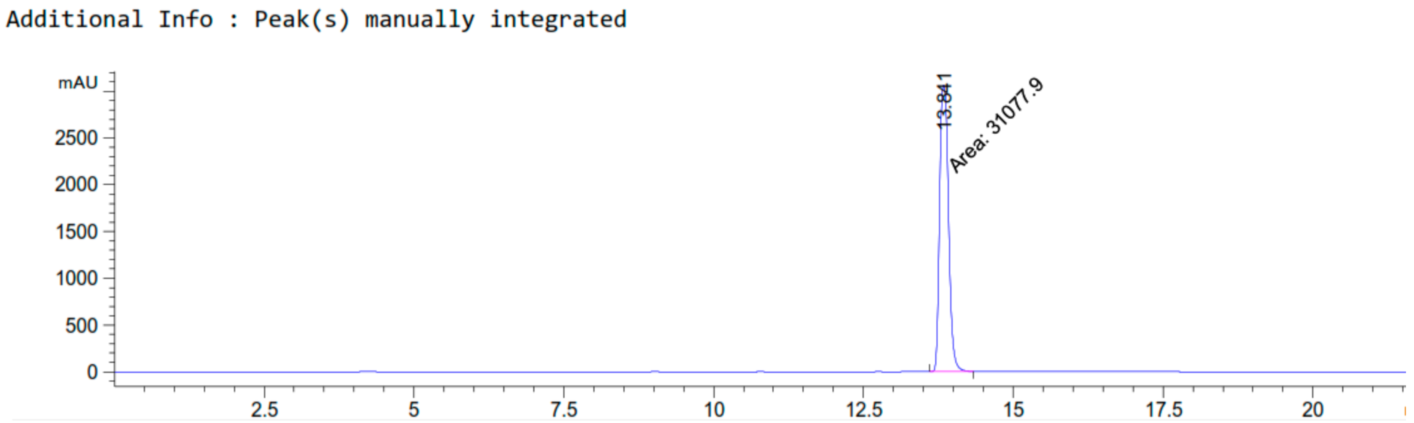

Figure S1. Chromatogram of caffeine standard at 300ppm

Chromatogram of standard obtained after the UHPLC analysis

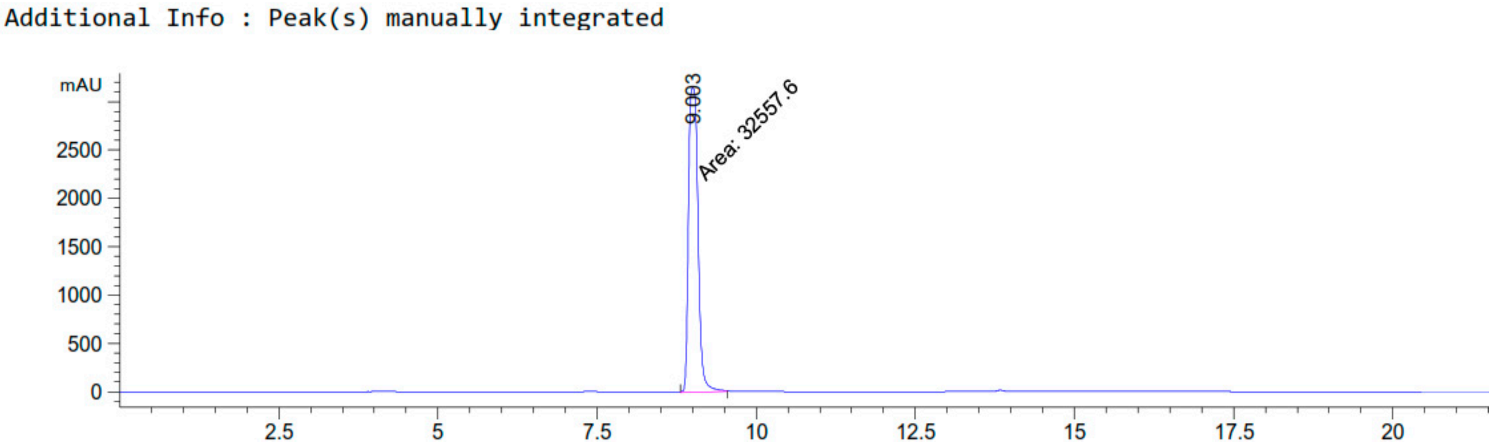

Figure S2. Chromatogram of theobromine standard at 300ppm

Chromatogram of standard obtained after the UHPLC analysis

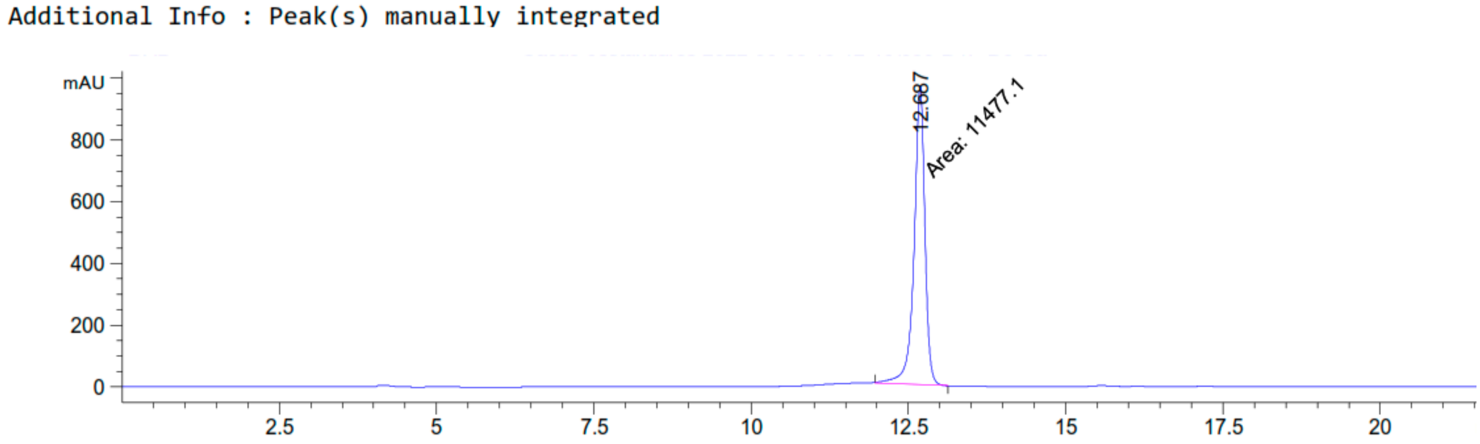

Figure S3. Chromatogram of catechin standard at 300ppm

Chromatogram of standard obtained after the UHPLC analysis

Additional Info : Peak(s) manually integrated

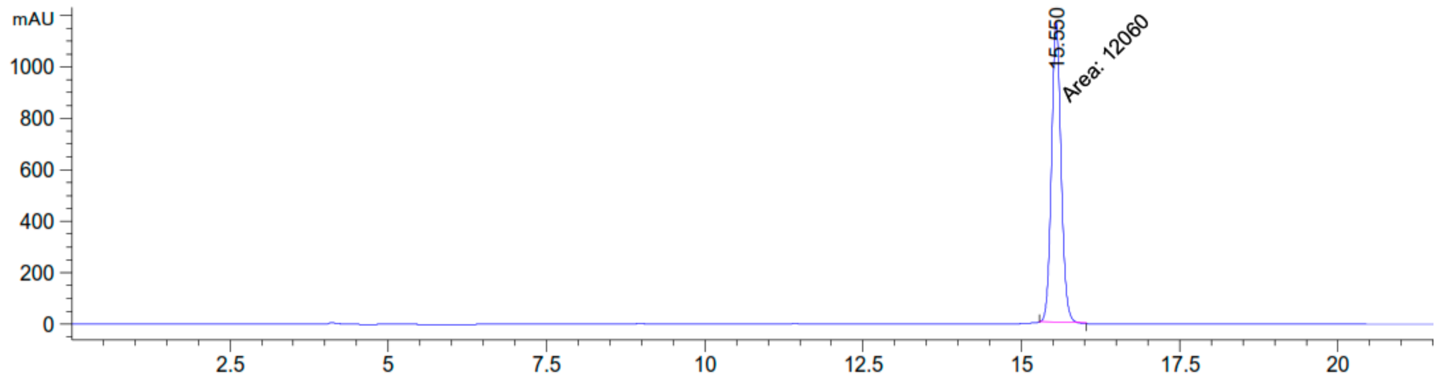

Figure S4. Chromatogram of epicatechin standard at 300ppm

Chromatogram of standard obtained after the UHPLC analysis

Table S3: Concentration of main volatile compounds (μg/g) by GC-MS in cocoa beans during spontaneous fermentation.

		COPALLIN								TOLOPAMPA								GUADALUPE							
		0h	24h	48h	72h	96h	120h	144h	168h	0h	24h	48h	72h	96h	120h	144h	168h	0h	24h	48h	72h	96h	120h	144h	168h
Aldehydes																									
2-methyl-Butanal	Almond, Cocoa, Fermented, Hazelnut, Malt	1.933	15.133	0.000	0.000	8.026	10.475	10.949	9.864	8.840	2.216	6.833	4.006	4.970	0.666	7.131	1.608	4.371	5.524	11.248	5.811	8.746	7.726	8.739	3.760
3-methyl-Butanal	Malt, chocolate	16.241	29.556	9.870	0.000	35.344	39.685	40.797	53.151	10.893	3.869	23.904	19.652	20.266	5.086	31.746	5.500	8.098	0.000	22.214	22.469	33.615	29.857	32.139	11.766
Hexanal	Apple, Fat, Fresh, Green, Oil	6.629	9.817	0.000	0.000	0.000	0.000	0.000	0.000	0.000	3.225	0.000	0.000	0.000	0.000	0.000	0.000	15.499	3.819	0.000	0.000	0.000	0.000	0.000	0.000
Nonanal	Fatty, Floral, Green, Lemon	0.000	0.000	0.000	0.000	0.000	0.000	0.000	0.000	0.000	0.000	0.000	0.000	0.000	0.000	0.000	0.000	0.000	0.000	0.000	0.000	0.000	0.000	0.000	1.959
Benzaldehyde	Bitter Almond, Burnt Sugar, Cherry, Malt, Roasted Pepper	0.000	0.000	0.000	0.000	0.000	0.000	0.000	19.342	0.000	0.000	0.000	2.632	0.000	0.000	2.933	0.000	0.000	0.000	0.000	8.641	7.890	0.000	7.057	0.000
Benzeneacetaldehyde	Berry, Geranium, Honey, Walnut, Spicy	16.520	35.024	87.550	53.291	119.904	73.100	98.505	78.281	19.394	25.441	145.572	82.140	90.764	13.823	88.953	25.453	9.669	49.768	98.661	99.291	120.490	123.960	70.371	62.413
Ketones																									
2-Pentanone	fruit, spicy	44.577	63.374	40.093	7.192	0.000	13.589	17.316	13.558	86.953	17.987	37.929	22.516	11.643	4.890	20.624	23.574	33.546	28.085	32.154	14.486	9.351	10.699	2.302	9.065
2,3-Butanedione	Butter, Pastry, Yeast	0.000	0.000	0.000	0.000	0.000	2.551	0.000	4.870	0.000	0.000	0.000	0.000	0.000	0.000	7.949	9.129	0.000	0.000	0.000	0.000	0.000	1.904	2.302	4.500
2-Heptanone	Blue Cheese, Fruit, Green, Nut, Spices	20.946	54.680	25.783	24.988	48.657	49.769	51.197	54.234	40.997	34.431	74.687	49.688	43.232	24.433	76.845	93.454	176.059	186.112	63.336	92.704	91.172	80.525	107.608	64.129
3-methyl-2-Butanone	flavoring agents	0.000	0.000	0.000	0.000	14.458	0.000	0.000	0.000	0.000	0.000	0.000	0.000	0.000	0.000	0.000	0.000	0.000	0.000	0.000	0.000	0.000	0.000	0.000	0.000
Acetoin	Butter, Creamy, Green Pepper	0.000	46.895	16.136	11.894	91.353	55.781	37.540	42.042	65.521	47.961	208.567	85.416	146.057	25.680	164.411	181.607	0.000	0.000	12.883	24.465	34.943	82.654	62.719	121.233
2-Nonanone	Fragrant, Fruit, Green, Hot Milk	2.328	0.000	0.000	0.000	7.809	0.000	0.000	0.000	0.000	11.222	0.000	9.707	0.000	3.717	1.747	6.051	17.774	51.558	0.000	20.306	4.482	23.690	7.675	4.471
Acetophenone	Almonds, Flower, Meat, Must	10.669	0.000	16.716	0.000	0.000	9.485	6.111	0.000	29.224	8.024	14.030	7.960	6.601	0.000	5.528	9.323	10.316	27.525	30.745	15.320	12.455	9.616	8.127	8.523
Alcohols																									
2-Butanol	Flavoring agents	0.000	0.000	0.000	0.000	0.000	0.000	0.000	0.000	0.000	0.000	0.000	0.000	0.000	0.000	0.000	0.000	1.592	0.000	0.000	0.000	0.000	0.000	0.000	0.000
Ethanol	Alcoholic	29.862	1469.252	823.368	1273.493	763.391	3.864	0.000	0.000	371.320	749.370	2674.538	1633.579	537.433	3.358	0.000	0.000	17.118	1842.631	2231.980	1999.211	1950.038	6.115	4.986	0.000
2-methyl-3-Buten-2-ol	flavoring agents	17.275	16.450	10.274	4.625	0.000	0.000	0.000	0.000	20.931	5.278	16.564	9.742	6.045	0.000	2.610	6.193	10.274	17.743	14.421	12.028	4.277	5.022	0.000	0.000
2-methyl-1-Propanol	Apple, Bitter, Cocoa, Wine	0.000	12.597	11.435	7.074	6.558	1.629	3.183	1.198	0.000	6.821	8.890	4.465	0.000	0.000	0.000	3.821	0.000	10.385	18.193	12.180	4.394	4.554	0.000	1.291
3-methyl-2-Butanol	flavoring agents	1.972	0.000	0.000	0.000	0.000	0.000	0.000	0.000	0.000	0.000	0.000	0.000	0.000	9.826	0.000	0.000	0.000	0.000	0.000	0.000	0.000	0.000	0.000	0.000
1-Butanol, 3-methyl-, acetate	Apple, Banana, Glue, Pear	0.000	0.000	23.339	21.587	123.729	45.095	80.990	21.806	0.000	2.840	31.739	55.327	123.488	25.449	72.429	73.524	0.000	0.000	0.000	79.849	44.965	102.785	15.152	38.059

2-Pentanol	Fusel Oil, Green	90.829	152.710	89.759	55.957	61.116	26.667	36.742	14.231	131.358	64.526	118.642	66.272	50.663	0.000	22.699	61.107	149.421	238.480	192.461	128.617	86.635	59.594	26.072	29.504
3-methyl-1-Butanol	Burnt, Cocoa, Floral, Malt	0.000	91.910	81.846	95.038	112.620	33.368	56.388	32.531	12.448	44.167	114.351	65.540	53.788	7.914	20.051	54.636	0.000	154.334	227.827	170.781	159.839	64.169	31.923	28.898
2-Hexanol		1.898	3.900	0.000	0.000	0.000	0.000	0.000	0.000	4.202	2.297	0.000	1.576	0.000	0.000	0.000	0.000	4.085	5.233	4.753	3.547	3.498	0.000	0.000	0.000
2-Heptanol	Citrus, Earth, Fried, Mushroom, Oil	53.864	227.741	38.473	71.760	188.771	69.141	70.254	44.670	67.515	75.136	128.344	91.270	86.117	24.226	55.262	122.718	362.734	553.651	177.042	307.717	248.461	149.647	106.667	104.505
2-Octanol	Fat, Fungus	0.000	0.000	0.000	0.000	0.000	0.000	0.000	0.000	0.000	0.000	0.000	0.000	0.000	0.000	0.000	0.000	0.000	3.766	0.000	0.000	0.000	0.000	0.000	0.000
2-Nonanol	Cucumber	0.000	8.539	0.000	0.000	11.147	0.000	0.000	0.000	0.000	3.917	0.000	3.836	0.000	1.948	0.000	4.899	11.932	60.848	6.152	19.118	2.901	16.139	7.946	3.974
2,3-Butanediol	flavoring agents	0.000	5.913	5.315	14.625	113.669	39.460	36.630	31.454	0.000	0.000	315.180	36.675	42.307	0.000	51.447	94.686	0.000	17.205	8.764	19.842	208.075	115.759	48.157	73.633
Benzyl alcohol	Boiled Cherries, Moss, Toast, Rose	0.000	0.000	0.000	0.000	0.000	0.000	0.000	0.000	0.000	0.000	0.000	0.000	0.000	0.000	0.000	0.000	47.186	0.000	0.000	0.000	0.000	0.000	0.000	0.000
Phenylethyl Alcohol	Fruit, Honey, Lilac, Rose, Wine	8.366	14.399	32.887	0.000	89.182	34.879	46.197	38.893	0.000	15.222	80.938	29.036	39.240	6.137	23.218	50.106	0.000	45.945	46.526	43.736	106.852	73.615	32.821	37.177
1-propanol	Alcohol, Caramel, Pungent	0.000	5.159	5.662	0.000	0.000	0.000	0.000	0.000	0.000	0.000	0.000	0.000	0.000	0.000	0.000	0.000	0.000	0.000	0.000	0.000	0.000	0.000	0.000	0.000
Esters																									
Isobutyl acetate	Apple, Banana, Floral, Grass	0.000	0.000	1.969	0.000	10.824	1.439	3.948	0.000	0.000	0.000	3.694	6.410	9.553	2.118	3.363	4.555	0.000	0.000	0.000	5.142	2.542	5.370	0.000	1.091
Acetic acid, butyl ester	Apple, Banana, Glue, Pungent	0.000	0.000	0.000	0.000	4.200	0.000	0.000	1.936	0.000	0.000	4.452	0.000	0.000	0.000	0.000	0.000	0.000	0.000	0.000	1.233	3.256	0.000	1.572	1.698
2-Pentanol, acetate	Fruit	0.000	0.000	6.518	11.545	17.130	17.090	23.294	4.531	0.000	1.887	12.095	16.362	33.035	8.347	22.367	40.800	0.000	0.000	0.000	14.968	16.051	28.663	5.393	10.333
Hexanoic acid, ethyl ester	Apple peel, brandy, bubble gum, overripe fruit, pineapple	0.000	0.000	0.000	0.000	9.245	0.000	0.000	0.000	0.000	0.000	0.000	2.715	5.251	0.000	0.000	0.000	0.000	0.000	0.000	14.905	4.978	4.330	0.000	1.913
Propanoic acid, 2-methyl-, ethyl ester	flavoring agents	0.000	0.000	1.822	0.000	0.000	0.000	0.000	0.000	0.000	0.000	0.000	0.000	0.000	0.000	0.000	0.000	0.000	0.000	0.000	0.000	0.000	0.000	0.000	0.000
Propanoic acid, 2-hydroxy-, ethyl ester	cheese, floral, fruity, spicy, rubber	0.000	0.000	0.000	10.450	0.000	0.000	0.000	0.000	0.000	0.000	0.000	4.436	0.000	0.000	0.000	0.000	0.000	0.000	14.171	0.000	24.013	0.000	0.000	0.000
Acetoin acetate	Fruit	0.000	0.000	0.000	0.000	0.000	0.000	0.000	0.000	0.000	0.000	0.000	0.000	4.765	1.608	3.314	10.107	0.000	0.000	0.000	0.000	0.000	0.000	0.000	4.258
Octanoic acid, ethyl ester	Apricot, Brandy, Fat, Floral, Pineapple	0.000	0.000	0.000	0.000	0.000	0.000	0.000	0.000	0.000	0.000	0.000	0.000	0.000	0.000	0.000	0.000	0.000	0.000	3.953	0.000	7.793	0.000	0.000	0.000
Acetic acid, 2-phenylethyl ester	flower, honey, rose	0.000	0.000	0.000	0.000	0.000	0.000	5.465	0.000	0.000	0.000	0.000	4.779	13.145	2.748	6.880	4.636	0.000	0.000	0.000	0.000	0.000	12.562	0.000	8.516
Hexadecanoic acid, ethyl ester	Wax	0.000	0.000	0.000	0.000	0.000	0.000	0.000	0.000	0.000	0.000	0.000	0.000	0.000	0.000	0.000	0.000	0.000	0.000	0.000	1.459	1.653	0.000	0.000	0.551
2-Heptanol, acetate	Flavoring agents	0.000	0.000	0.000	5.668	0.000	3.418	0.000	0.000	0.000	2.262	0.000	0.000	0.000	0.000	0.000	0.000	0.000	0.000	0.000	0.000	0.000	0.000	0.000	0.000
Pentanoic acid, 2-hydroxy-4-methyl-, methyl ester	Fruit	0.000	0.000	0.000	0.000	0.000	0.000	0.000	0.000	0.000	0.000	0.000	0.000	0.000	0.000	2.089	0.000	0.000	0.000	0.000	0.000	0.000	0.000	0.000	0.000
Benzeneacetic acid, ethyl ester	Floral, Fruit, Honey, Rose	0.000	0.000	0.000	0.000	0.000	2.711	0.000	0.000	0.000	0.000	0.000	0.000	5.181	0.000	0.000	0.000	0.000	0.000	0.000	0.000	0.000	0.000	0.000	0.000

Ethyl Acetate	Aromatic, Brandy, Grape	0.000	0.000	0.000	98.395	0.000	0.000	0.000	0.000	0.000	0.000	0.000	0.000	0.000	0.000	0.000	0.000	0.000	0.000	0.000	0.000	0.000	0.000	0.000	0.000
Ethyl Oleate	Dairy products	0.000	0.000	0.000	0.000	0.000	0.000	0.000	0.000	0.000	0.000	0.000	0.000	0.000	0.000	0.000	0.000	0.000	0.000	0.000	0.000	1.068	0.000	0.000	0.000
Acids																									
Acetic acid	acid, fruit, pungent, sour, vinega	4.359	24.459	29.884	104.892	1847.125	242.143	516.434	142.485	26.268	0.000	1318.287	570.058	1241.960	172.023	386.144	894.381	0.000	31.135	28.849	217.822	971.303	1699.416	472.169	715.373
2-methyl-Propanoic acid	Burnt, Butter, Cheese, Sweat	0.000	0.000	0.000	0.000	0.000	27.503	32.857	21.317	0.000	0.000	0.000	0.000	0.000	4.454	32.695	33.005	0.000	0.000	0.000	0.000	0.000	45.872	41.995	39.543
3-methyl-Butanoic acid	cheese, spicy	0.000	0.000	0.000	0.000	18.195	73.299	93.240	47.259	0.000	0.000	0.000	0.000	7.791	11.442	65.820	68.446	0.000	0.000	0.000	0.000	9.508	135.061	110.943	114.550
Octanoic acid	Cheese, Fat, Grass, Oil	0.772	0.000	2.195	0.000	3.149	1.288	0.000	1.301	0.000	0.000	0.000	0.000	0.000	0.000	0.000	0.000	0.000	0.000	0.000	0.000	0.000	2.577	0.000	0.000
Terpenoids and Terpenes																									
D-Limonene	citrus, mint	0.000	0.000	0.000	0.000	0.000	0.000	0.000	2.120	0.000	0.000	0.000	0.000	0.000	0.000	0.000	4.149	0.000	0.000	0.000	0.000	0.000	0.000	0.000	0.000
trans-Linalool oxide	flavoring agent	5.670	0.000	0.000	0.000	0.000	4.983	6.069	3.569	4.144	1.875	17.325	5.272	5.723	1.027	3.313	8.209	0.000	6.752	0.000	4.105	4.540	0.000	0.000	3.317
Linalool	coriander, floral, lavender, lemon, rose	177.726	19.117	26.262	93.116	30.360	74.975	89.087	97.665	26.643	19.329	62.756	16.609	26.130	6.997	23.935	34.959	8.357	39.989	7.204	27.246	27.109	11.903	9.941	13.741
Hydrocarbons																									
Toluene	caramel, synthetic (off-flavor)	0.000	0.000	0.000	0.000	0.000	0.000	1.806	1.990	4.267	0.000	0.000	0.000	0.000	0.000	0.000	3.603	0.000	0.000	0.000	0.000	0.000	2.632	0.000	0.000
beta.-Myrcene	balsamic, fruit, geranium, herb, must	0.000	5.897	0.000	0.000	0.000	21.556	4.287	17.468	0.000	3.520	0.000	0.000	0.000	0.000	6.261	0.000	0.000	9.031	0.000	12.029	0.000	0.000	2.872	0.000
Styrene	Sweet balsam, floral plastic (off-flavor)	0.000	0.000	0.000	0.000	7.991	0.000	4.788	5.177	5.555	0.000	9.724	0.000	0.000	0.000	0.000	8.888	0.000	0.000	0.000	0.000	0.000	9.087	0.000	0.000
trans-.beta.-Ocimene	Floral	0.000	6.402	0.000	0.000	0.000	28.031	8.087	22.401	0.000	3.434	0.000	0.000	0.000	0.000	8.743	0.000	0.000	6.135	0.000	0.000	0.000	0.000	0.000	0.000
Acetal																									
2,4,5-trimethyl-1,3-Dioxolane	Indicator of oxidation	0.000	0.000	4.573	0.000	0.000	3.084	2.468	1.643	0.000	0.000	0.000	126.990	0.000	0.000	2.414	4.086	0.000	0.000	0.000	0.000	0.000	2.047	2.572	1.979

Table S4: Concentration of main volatile compounds (μg/g) by GC-MS in cocoa beans during fermentation with starter culture

Compounds	Observation	COPALLIN								TOLOPAMPA								GUADALUPE							
		0h	24h	48h	72h	96h	120h	144h	168h	0h	24h	48h	72h	96h	120h	144h	168h	0h	24h	48h	72h	96h	120h	144h	168h
Aldehydes																									
2-methyl-Butanal	Almond, Cocoa, Fermented, Hazelnut, Malt	10.030	5.419	0.000	0.000	7.987	3.892	1.059	8.169	1.762	9.317	1.038	8.789	5.817	12.067	6.316	17.562	3.334	4.996	0.000	3.904	7.300	1.626	7.579	6.395
3-methyl-Butanal	Malt, chocolate	12.852	9.971	0.000	1.352	35.483	18.743	4.924	35.658	2.706	17.361	0.000	37.599	30.253	58.668	30.774	76.318	0.000	5.707	0.949	0.000	34.197	7.096	14.262	21.720
Hexanal	Apple, Fat, Fresh, Green, Oil	5.940	21.995	0.000	0.000	0.000	0.000	0.000	0.000	0.000	6.388	0.000	0.000	0.000	0.000	0.000	0.000	0.000	4.753	0.000	0.000	0.000	0.000	0.000	0.000
Benzaldehyde	Bitter Almond, Burnt Sugar, Cherry, Malt, Roasted Pepper	0.000	0.000	0.000	0.000	8.395	0.000	0.000	0.000	0.000	0.000	0.000	11.661	0.000	0.000	0.000	0.000	0.000	0.000	0.000	34.422	0.000	0.000	0.000	20.946
Benzeneacetaldehyde	Berry, Geranium, Honey, Walnut, Spicy	22.105	27.861	123.685	19.405	85.981	63.252	11.595	91.263	14.368	33.870	28.408	112.798	139.429	162.805	126.965	275.746	6.614	26.537	8.874	66.915	110.643	21.308	57.780	64.711
Ketones																									
2-Pentanone	Fruit, spicy	41.644	38.284	37.145	11.487	26.255	18.839	3.098	14.281	4.945	39.686	10.356	8.696	10.767	28.728	24.978	26.578	24.536	31.079	3.959	10.064	15.118	2.884	6.438	10.201
2,3-Butanedione	Butter, Pastry, Yeast	0.000	0.000	0.000	0.000	0.000	0.000	0.000	10.095	0.000	0.000	0.000	3.358	0.000	10.861	0.000	9.792	0.000	0.000	0.000	0.000	0.000	0.000	0.000	4.398
2-Heptanone	Blue Cheese, Fruit, Green, Nut, Spices	42.410	84.287	49.584	24.375	68.877	49.868	16.637	119.486	137.431	51.288	38.865	52.954	43.731	131.024	99.470	86.948	16.208	208.904	42.915	91.558	106.280	30.483	69.812	115.946
Acetoin	Butter, Creamy, Green Pepper	0.000	24.487	92.123	17.098	59.090	58.653	11.356	150.257	43.609	28.416	27.158	88.587	147.227	198.513	161.465	180.952	0.000	11.187	7.950	59.012	109.139	51.751	68.748	176.843
2-Nonanone	Fragrant, Fruit, Green, Hot Milk	3.614	4.383	0.000	0.000	0.000	3.902	0.000	8.855	8.955	0.000	9.527	0.000	5.137	34.476	36.245	0.000	0.000	53.485	4.723	68.403	0.000	0.000	8.289	4.387
Acetophenone	Almonds, Flower, Meat, Must	6.849	7.355	20.873	0.000	0.000	4.516	0.000	9.092	3.887	10.276	0.000	14.611	10.397	9.275	0.000	13.666	5.571	16.988	0.000	0.000	16.041	0.000	6.250	14.592
2-Octanone	Fat, Fragrant, Mildew	0.000	0.000	0.000	0.000	0.000	0.000	0.000	0.000	1.686	0.000	0.000	0.000	0.000	0.000	0.000	0.000	0.000	0.000	0.000	0.000	0.000	0.000	0.000	0.000
Alcohols																									
2-Butanol	flavoring agents	0.000	0.000	0.000	0.000	0.000	0.000	0.000	0.000	0.000	0.000	0.000	0.000	0.000	0.000	0.000	7.375	0.000	0.000	0.000	0.000	0.000	0.000	0.000	0.000
Ethanol	Alcoholic	0.000	826.639	3063.627	325.958	1897.658	0.000	0.000	12.803	183.345	1848.775	128.067	335.173	400.244	27.974	220.215	0.000	21.298	926.861	231.748	2628.314	778.737	12.306	0.000	0.000
2-methyl-3-Buten-2-ol	Flavoring agents	16.817	8.277	29.085	3.161	13.144	4.480	0.000	0.000	5.487	11.638	3.681	5.383	6.835	8.667	12.446	13.738	7.902	6.974	0.000	9.874	9.764	0.000	0.000	2.710
2-methyl-1-Propanol	Apple, Bitter, Cocoa, Wine	0.000	8.155	15.429	2.499	8.343	3.056	0.000	2.721	0.000	7.701	2.560	3.296	0.000	4.723	6.537	5.596	0.000	1.913	2.705	12.301	11.994	1.539	2.785	2.564
3-methyl-2-Butanol	Flavoring agents	0.000	0.000	0.000	33.506	0.000	0.000	7.490	30.797	0.000	0.000	0.000	0.000	0.000	0.000	0.000	0.000	0.000	0.000	0.000	0.000	0.000	0.000	47.355	0.000
1-Butanol, 3-methyl-, acetate	Apple, Banana, Glue, Pear	0.000	0.000	34.491	60.470	181.737	75.114	32.195	129.672	0.000	2.951	50.359	86.046	125.856	111.891	144.989	143.230	0.000	0.000	28.524	54.783	116.450	64.751	89.977	69.209
2-Pentanol	Fusel Oil, Green	197.424	77.156	131.697	0.000	109.764	45.815	0.000	0.000	38.661	95.195	34.243	45.565	41.550	59.678	72.648	75.010	99.138	111.810	21.088	127.175	103.998	13.875	0.000	43.249
3-methyl-1-Butanol	Burnt, Cocoa, Floral, Malt	0.000	56.330	137.345	48.986	170.378	50.689	11.004	65.744	12.861	66.917	29.288	44.053	43.466	41.990	55.664	45.652	0.000	47.373	52.765	199.247	224.856	23.432	79.713	73.071
2-Hexanol	Fruity	4.742	0.000	0.000	0.000	0.000	0.000	0.000	0.000	1.921	0.000	0.000	0.000	0.000	0.000	0.000	0.000	2.279	0.000	0.000	0.000	0.000	0.000	0.000	0.000
2-Heptanol	Citrus, Earth, Fried, Mushroom, Oil	102.120	168.264	116.025	54.225	158.590	74.202	30.915	161.907	198.350	56.192	68.423	78.872	62.144	152.892	137.868	95.638	34.930	359.090	95.909	622.093	280.714	40.283	144.321	168.523
2-Octanol	Fat, Fungus	0.000	0.000	0.000	0.000	0.000	0.000	0.000	0.000	0.000	0.000	0.000	0.000	0.000	0.000	0.000	0.000	0.000	0.000	0.000	10.429	0.000	0.000	0.000	0.000
2-Nonanol	Cucumber	0.000	0.000	0.000	0.000	0.000	0.000	0.000	6.810	2.401	0.000	5.824	0.000	0.000	18.358	20.534	0.000	0.000	32.595	2.012	118.419	5.555	0.000	4.571	5.413
2,3-Butanediol	Flavoring agents	0.000	5.581	119.779	0.000	170.262	79.651	12.442	102.667	0.000	19.094	0.000	48.177	44.033	91.170	158.492	229.637	0.000	16.828	4.525	112.644	53.506	25.183	27.881	149.207
Benzyl alcohol	Boiled Cherries, Moss, Toast, Rose	0.000	0.000	0.000	0.000	0.000	0.000	0.000	0.000	0.000	10.280	0.000	0.000	0.000	0.000	0.000	0.000	0.000	13.051	0.000	59.192	0.000	0.000	0.000	0.000
Phenylethyl Alcohol	Fruit, Honey, Lilac, Rose, Wine	0.000	14.473	37.458	10.532	68.747	31.133	8.931	66.396	3.472	16.890	10.004	38.239	33.105	33.913	48.924	50.939	0.000	16.921	14.326	68.932	99.636	17.472	101.286	88.759
1-Pentanol	Balsamic, fruit, green, spicy, yeast	0.000	4.201	0.000	0.000	0.000	0.000	0.000	0.000	0.000	0.000	0.000	0.000	0.000	0.000	0.000	0.000	0.000	0.000	0.000	0.000	0.000	0.000	0.000	0.000
Esters																									

Isobutyl acetate	Apple, Banana, Floral, Grass	0.000	0.000	3.621	5.367	10.678	7.442	1.591	3.791	0.000	0.000	5.921	4.361	11.715	7.046	14.342	12.852	0.000	0.000	2.083	0.000	5.118	2.409	3.488	2.152
Acetic acid, butyl ester	Apple, Banana, Glue, Pungent	0.000	0.000	3.907	0.000	6.775	1.728	0.000	0.000	0.000	0.000	0.000	0.000	0.000	0.000	0.000	0.000	0.000	0.000	0.000	0.000	4.444	0.000	0.000	4.618
2-Pentanol, acetate	Fruit	0.000	0.000	9.798	10.806	35.604	15.403	6.150	37.244	0.000	2.041	14.556	34.467	36.728	72.593	58.397	71.138	0.000	0.000	2.883	8.156	27.374	11.667	21.145	13.265
Hexanoic acid, ethyl ester	Apple peel, brandy, bubble gum, overripe fruit, pineapple	0.000	0.000	6.065	0.000	0.000	0.000	0.000	0.000	0.000	0.000	0.000	5.216	0.000	0.000	0.000	0.000	0.000	0.000	0.000	0.000	13.426	3.372	0.000	7.057
Propanoic acid, 2-hydroxy-, ethyl ester	cheese, floral, fruity, spicy, rubber	0.000	0.000	0.000	0.000	16.003	0.000	0.000	0.000	0.000	0.000	0.000	3.145	0.000	0.000	0.000	0.000	0.000	0.000	0.000	41.371	15.542	0.000	0.000	0.000
Acetoin acetate	Fruit	0.000	0.000	0.000	0.000	0.000	0.000	0.000	0.000	0.000	0.000	0.000	4.767	6.454	11.091	7.839	11.461	0.000	0.000	0.000	0.000	0.000	0.000	0.000	2.884
1-Methoxy-2-propyl acetate	Ester identified in CB	0.000	0.000	0.000	0.000	0.000	0.000	0.000	0.000	0.000	0.000	0.000	0.000	3.469	0.000	8.305	6.274	0.000	0.000	0.000	0.000	0.000	0.000	3.778	5.684
Acetic acid, 2-phenylethyl ester	flower, honey, rose	0.000	0.000	0.000	3.379	7.910	3.130	3.784	0.000	0.000	0.000	4.316	10.865	14.526	6.369	18.890	12.795	0.000	0.000	0.000	0.000	0.000	4.145	0.000	0.000
Hexadecanoic acid, ethyl ester	Wax	0.000	0.000	0.000	0.000	0.000	0.000	0.000	0.000	0.000	0.000	0.000	0.813	1.137	0.000	0.000	0.000	0.000	0.000	0.000	2.102	0.000	0.000	2.657	0.000
2-Octanol acetate	citrus (orange)	0.000	0.000	0.000	0.000	0.000	0.000	0.000	0.000	0.000	0.000	0.000	0.000	0.000	0.000	0.000	6.299	0.000	0.000	0.000	0.000	0.000	0.000	0.000	0.000
1,2-Propanediol, diacetate	Fruit	0.000	0.000	0.000	0.000	0.000	0.000	0.000	0.000	0.000	0.000	0.000	2.479	0.000	0.000	0.000	0.000	0.000	0.000	0.000	0.000	0.000	0.000	0.000	0.000
Benzeneacetic acid, ethyl ester	Floral, Fruit, Honey, Rose	0.000	0.000	0.000	0.000	6.522	0.000	0.000	0.000	0.000	0.000	0.000	4.408	5.005	0.000	4.934	0.000	0.000	0.000	0.000	0.000	0.000	0.000	0.000	0.000
Acids																									
Acetic acid	Acid, fruit, pungent, sour, vinegar	6.169	21.369	669.354	180.072	1538.792	700.360	158.411	590.989	0.000	52.478	301.771	774.888	1360.353	1676.702	2723.658	2452.167	0.000	27.621	167.668	628.150	1703.457	390.735	754.126	1394.497
2-methyl-Propanoic acid	Burnt, Butter, Cheese, Sweat	0.000	0.000	0.000	0.000	0.000	33.665	8.237	64.297	0.000	0.000	0.000	2.659	4.017	34.232	19.179	48.367	0.000	0.000	0.000	0.000	4.426	10.704	45.395	60.782
3-methyl-Butanoic acid	cheese, spicy	0.000	0.000	0.000	0.000	0.000	88.288	29.373	178.271	0.000	0.000	0.000	5.632	6.221	56.946	33.756	101.646	0.000	0.000	0.000	0.000	26.032	39.265	126.963	200.954
Octanoic acid	Cheese, Fat, Grass, Oil	0.000	0.000	0.000	1.822	0.000	1.173	0.523	2.553	0.000	0.000	0.000	0.000	0.000	0.000	0.000	0.000	0.000	0.000	0.000	4.430	0.000	0.952	2.523	0.000
n-Hexadecanoic acid	Flavoring agents	0.000	0.000	10.067	1.199	0.000	0.000	0.525	0.000	0.000	0.000	2.407	0.000	0.000	0.000	0.000	0.000	0.925	1.991	0.700	0.000	0.000	1.322	0.000	0.000
diethyl-Acetic acid	Fruit	0.000	0.000	0.000	0.000	0.000	0.000	0.000	3.931	0.000	0.000	0.000	0.000	0.000	0.000	0.000	0.000	0.000	0.000	0.000	0.000	0.000	0.000	0.000	0.000
Terpenoids and terpenes																									
D-Limonene	citrus, mint	0.000	0.000	0.000	0.000	0.000	0.000	0.000	0.000	0.000	0.000	0.000	0.000	0.000	0.000	0.000	0.000	0.000	0.000	0.000	0.000	0.000	0.000	0.000	5.203
trans-Linalool oxide	Flavoring agent	2.962	0.000	14.470	0.000	6.429	4.980	0.000	6.699	5.334	0.000	0.000	7.501	4.548	6.364	9.328	8.356	0.000	0.000	0.000	0.000	6.662	0.000	3.960	5.984
Linalool	Coriander, floral, lavender, lemon, rose	7.195	5.695	223.784	17.014	121.159	17.492	17.191	45.398	31.203	3.853	16.111	32.651	42.636	107.878	67.750	24.608	24.146	4.636	11.520	289.716	31.739	6.631	25.461	45.018
Furans, furanones, pyrans, pyrones																									
1-(2-furanyl)-Ethanone	Balsamic, Cocoa, Coffee	0.000	0.000	0.000	0.000	0.000	0.000	0.000	0.000	0.000	0.000	0.000	0.000	0.000	3.747	0.000	0.000	0.000	0.000	0.000	0.000	0.000	0.000	0.000	0.000
Hydrocarbons																									
Toluene	Caramel, synthetic (off-flavor)	2.211	0.000	0.000	0.000	0.000	1.516	0.000	4.197	0.000	0.000	0.000	0.000	0.000	0.000	3.179	0.000	0.000	0.000	0.000	0.000	0.000	0.000	1.787	3.686
beta.-Myrcene	Balsamic, fruit, geranium, herb, must	0.000	0.000	7.092	0.000	62.976	0.000	0.000	0.000	0.000	0.000	0.000	0.000	16.223	15.009	5.034	0.000	3.968	8.730	0.000	146.569	12.246	0.000	0.000	2.454
Styrene	Sweet balsam, floral plastic (off-flavor)	0.000	0.000	8.046	0.000	0.000	4.845	0.000	5.551	2.175	0.000	0.000	0.000	0.000	0.000	9.501	11.766	0.000	0.000	0.000	0.000	0.000	0.000	0.000	13.339
trans-.beta.-Ocimene	Floral	0.000	0.000	0.000	0.000	84.361	0.000	0.926	0.000	0.000	0.000	0.000	0.000	23.222	13.294	0.000	0.000	4.215	4.129	0.000	194.056	0.000	0.000	11.738	0.000
Acetal																									
2,4,5-trimethyl-1,3-Dioxolane		0.000	0.000	0.000	0.378	0.000	5.234	0.000	3.246	0.000	0.000	0.000	0.000	0.000	4.675	3.886	4.613	0.000	0.000	0.000	0.000	4.167	0.000	1.971	1.182

The roasting process and place of cultivation influence the volatile fingerprint of Criollo cocoa from Amazonas, Peru
